# Revisiting Supersaturation of a Biopharmaceutical Classification System IIB Drug: Evaluation via a Multi-Cup Dissolution Approach and Molecular Dynamic Simulation

**DOI:** 10.3390/molecules28196962

**Published:** 2023-10-07

**Authors:** Yanxiong Gan, Yaxin Xu, Xue Zhang, Huiling Hu, Wenke Xiao, Zheng Yu, Tao Sun, Jinming Zhang, Chuanbiao Wen, Shichao Zheng

**Affiliations:** 1 School of Intelligent Medicine, Chengdu University of Traditional Chinese Medicine, Chengdu 611137, China; ganyanxiong@cdutcm.edu.cn (Y.G.); suntao513@gmail.com (T.S.); 2 School of Pharmacy, China Pharmaceutical University, Nanjing 210009, China; 3 Jiangsu Hengrui Medicine Co., Ltd., Nanjing 210009, China; 4 State Key Laboratory of Southwestern Chinese Medicine Resources, School of Pharmacy, Chengdu University of Traditional Chinese Medicine, Chengdu 611137, Chinacdutcmzjm@126.com (J.Z.)

**Keywords:** supersaturation, precipitation inhibitor, dissolution model, molecular dynamics, in vitro–in vivo correlation

## Abstract

As a subclass of the biopharmaceutical classification system (BCS) class II, basic drugs (BCS IIB) exhibit pH-dependent solubility and tend to generate supersaturation in the gastrointestinal tract, leading to less qualified in vitro–in vivo correlation (IVIVC). This study aims to develop a physiologically based multi-cup dissolution approach to improve the evaluation of the supersaturation for a higher quality of IVIVC and preliminarily explores the molecular mechanism of supersaturation and precipitation of ketoconazole affected by Polyvinylpyrrolidone–vinyl acetate copolymer (PVPVA) and hydroxypropyl methyl-cellulose (HPMC). The concentration of ketoconazole in each cup of the dynamic gastrointestinal model (DGIM) was measured using fiber optical probes. Molecular interactions between ketoconazole and PVPVA or HPMC were simulated by Materials Studio. The results demonstrated that PVPVA and HPMC improved and maintained the supersaturation of ketoconazole. PVPVA exhibited superior precipitation inhibitory effect on ketoconazole molecule aggregation due to slightly stronger van der Waals forces as well as unique electrostatic forces, thereby further enhancing in vitro drug absorption, which correlated well with in vivo drug absorption. Compared with a conventional dissolution apparatus paddle method, the DGIM improved the mean prediction error through the IVIVC from 19.30% to 9.96%, reaching the qualification criteria. In conclusion, the physiologically based multi-cup dissolution approach enables improved evaluation of supersaturation in gastrointestinal transportation of BCS IIB drug ketoconazole, enabling screening screen precipitation inhibitors and achieving qualified IVIVC for drug formulation studies.

## 1. Introduction

Supersaturation is preliminarily recognized as a strategy for enhancing the oral bioavailability of poor solubility drugs [1]. Some immediate-release formulations can function as “springs” to rapidly release significant quantities of drug molecules into water, resulting in a concentration surpassing the equilibrium solubility and thus generating supersaturation. However, supersaturation is thermodynamically unstable. Precipitation inhibitors (PIs) are employed as a “parachute” to mitigate precipitation and prolong the state of supersaturation [2]. The increased concentration during this state allows for enhanced permeation of drug molecules across the intestinal membrane. Specifically, as a subclass of the biopharmaceutical classification system (BCS) class II, some weakly basic drugs (BCS IIB) can experience supersaturation, which is spontaneously generated since the pH shifts from that of the acidic gastric fluid to the intestinal fluids with increasing pH [3]. Due to the complex process of supersaturation and precipitation, an appropriate evaluation approach is crucial for screening PIs and achieving qualified in vitro–in vivo correlation (IVIVC) for supersaturable drug delivery systems.

United States Pharmacopeia Dissolution Apparatus II (USP II, paddle method) is widely employed as a compendial method for assessing the dissolution of oral solid formulations, which is similar to that described for Chinese, European, and Japanese Pharmacopeia. USP II is easy to apply due to the standardized provisions. However, when it comes to supersaturable drug delivery systems, this advantage hampers accurate simulation of dissolution in the gastrointestinal tract, leading to unsatisfied IVIVC [4]. Consequently, numerous researchers have proposed novel methods to evaluate the supersaturation and precipitation of drugs [5]. Mitra and Fadda developed a simulated stomach duodenum (SSD) model comprising a gastric cup with a volume of 50 mL and a duodenal cup with a volume of 30 mL [6]. Further, a jejunal cup was added to improve the gastrointestinal simulator (GIS) by Amidon’s research group [7,8,9]. Yasuhiro et al. [10] further introduced GIS-α as the first commercially available transfer-model dissolution system; however, it exhibited prediction errors (PEs) exceeding 51% (AUC) and 35% (C_max_). The specially designed devices are limited for wide application in the industry. Due to the lack of an absorptive phase, their precipitation rate might be overestimated, and supersaturation might be underestimated [11]. To address this issue, mouse intestinal perfusion [12] was combined with GIS, achieving level C IVIVC with R^2^ = 0.966 for C_max_. Subsequently, decanol [13]/octanol [14] were added to the jejunal cup, simulating absorption, and their PE reached 51% (AUC), 28% (C_max_)/20% (AUC), and 2.3% (C_max_), respectively. We preliminarily developed a biphasic GIS [15] by incorporating octanol as absorptive phases in both the duodenal cup and the jejunal cup. The in vitro absorption data obtained from the combined absorptive phases demonstrated a level A IVIVC (PE < 10%). However, the approach using beakers and magnetic stirring is low in commonality. Therefore, it is necessary to enhance the assessment approach for supersaturation, striking a balance between commonality and usability for the pharmaceutical industry.

As proposed in a recent review of the evaluation approaches about supersaturation [16], a comprehensive evaluation of the effect of precipitation inhibitors on supersaturation requires not only physiological in vitro dissolution to correlate with in vivo absorption but also molecular modeling techniques such as molecular dynamic simulation to investigate the underlying molecular mechanisms governing the interaction between precipitation inhibitors and drugs. For instance, Zhang et al. [17] employed molecular dynamic simulation to demonstrate that hydroxylpropylmethyl cellulose acetate succinate (HPMCAS) maintains loratadine supersaturation more effectively than polyvinylpyrrolidone-co-vinyl-acetate (PVPVA) by forming stronger interactions with drug nanoparticles at their surface. However, it should be noted that the ratio of molecules can influence the interaction energy between polymer and drug. Additionally, HPMCAS is an enteric polymer, which limits its ability in the supersaturation induced by the pH shift from the stomach to the intestine. Therefore, conducting molecular dynamic simulations following relevant experiments becomes crucial.

The primary aim of this research was to enhance the evaluation of supersaturation using a multi-cup dissolution approach based on USP II and elucidate the molecular mechanisms underlying supersaturation improvement facilitated by precipitation inhibitors. We developed a dynamic gastrointestinal model (DGIM) based on the theory of the biphasic GIS [15] but implemented it within the framework of USP II. Specifically, most operational parameters were aligned with those of biphasic GIS, except for the replacement of three beakers with dissolution cups and magnetic stirring with paddle stirring. For comparison, a conventional USP II method was also conducted, incorporating an absorptive phase through octanol addition and pH shift via simulated intestinal fluid dumping. In vivo prediction parameters obtained from both methods were employed to assess simulation accuracy. Ketoconazole, a commonly used BCS II B drug for supersaturation studies, was chosen as the model drug in this study. Polyvinylpyrrolidone–vinyl acetate copolymer (PVPVA) and hydroxypropyl methylcellulose (HPMC) were chosen as the model PIs. Additionally, to preliminarily explore the mechanism behind supersaturation and precipitation phenomena, molecular dynamic simulations were performed alongside the characterization of precipitates to investigate drug-PI molecular interactions.

## 2. Results and Discussions

### 2.1. pH-Dependent Solubility

**Figure 1:**
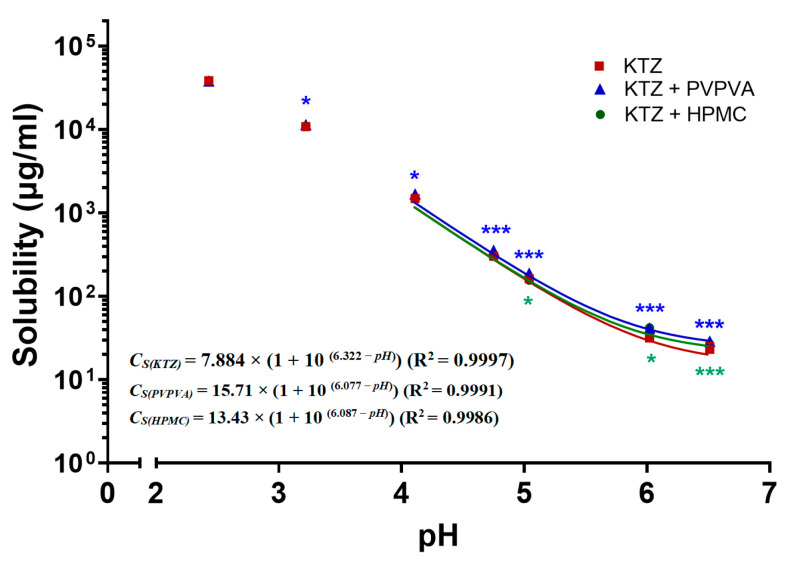
The pH–solubility of ketoconazole (KTZ) with polyvinylpyrrolidone–vinyl acetate copolymer (PVPVA) or hydroxypropyl methyl-cellulose (HPMC) in biorelevant fluids at 37 °C. The solid lines are the fitted pH–solubility curves through the modified Henderson–Hasselbalch equation. The data points are presented as the mean ± SD (*n* = 3). * for *p* < 0.05 and *** for *p* < 0.001, their color represent the same group as the symbols.

The thermodynamic solubility of ketoconazole was observed to decrease not only with an elevation in pH but was also affected by the presence of PVPVA and HPMC, as shown by the dots in Figure 1. Compared with the control group without PI, the PVPVA group significantly increased the equilibrium solubility of ketoconazole 1.06-, 1.14-, 1.18-, 1.17-, 1.32-, and 1.25-fold at pH values of 3.2, 4.0, 4.7, 5.0, 6.0, and 6.5; the HPMC group increased the equilibrium solubility of ketoconazole 1.33- and 1.16-fold at pH values of 6.0 and 6.5.

Then, in order to calculate the equilibrium solubility for the further degree of supersaturation, the Henderson–Hasselbalch equation was modified [18] to fit the above equilibrium solubility of ketoconazole at different pH:

*C_S_* = *C_U_* × (1 + 10^(*pKa*−*pH*)^) (1)

where *C_S_* represents the solubility, *C_U_* represents the concentration of unionized form, and *pK*_a_ represents the acid dissociation constants. After excluding the biased data from the pH values of 2.4 and 3.2, the fitted pH–solubility curves and the functions are shown in Figure 1. The *pK*_a_ in the functions was reduced from 6.322 to 6.077 and 6.087 by PVPVA and HPMC, respectively, which are close to the reported value of 6.5 in the phosphate buffer [19].

### 2.2. In Vitro Supersaturation–Precipitation and Absorption Assay

#### 2.2.1. Supersaturation–Precipitation and Absorption in the DGIM

**Figure 2:**
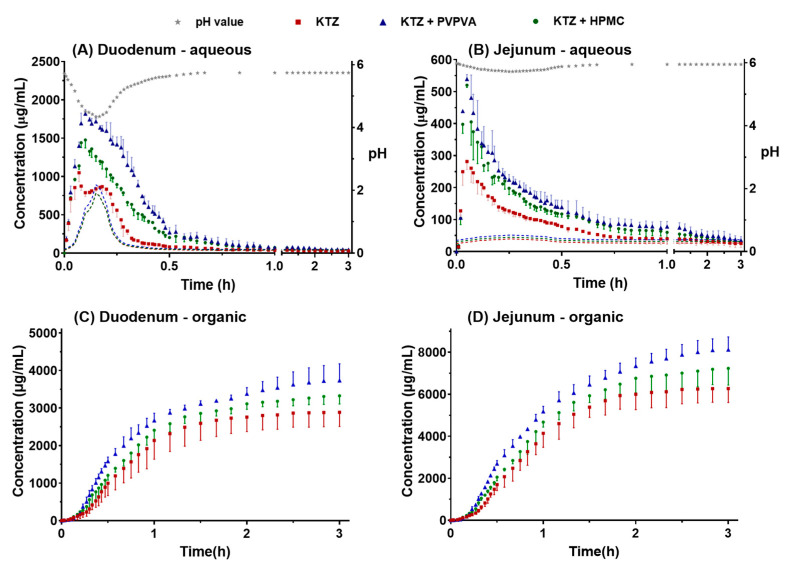
The profiles of the ketoconazole concentration–time observed in the aqueous phase in the duodenal cup (**A**), aqueous phase in the jejunal cup (**B**), organic phase in the duodenal cup (**C**), and organic phase in the jejunal cup (**D**). The solubility of ketoconazole in real-time pH was calculated and represented by dashed lines, which matched the color of corresponding data points. The data points are presented as the mean ± SD (*n* = 3).

The concentration of ketoconazole in each dissolution cup of the DGIM during the transfer is illustrated in Figure 2. As the pro-dissolved ketoconazole in the FaSSGF transported from the gastric cup to the duodenum cup, the concentration of ketoconazole of three groups in the aqua phase of the duodenal cup rapidly increased in the former 5 min but then soon decreased because the pH value in the aqua phase of the duodenal cup suffered from the large flux of FaSSGF in the beginning. Then, as the reducing rate of transfer and the constant secretion of concentrated FaSSIF, the pH value reverted to near 6 (Figure 2A). The equilibrium solubility of ketoconazole (the dashed lines shown in Figure 2A) was calculated according to the pH value and the fitted curves. Another important reason is that the supersaturation was generated in the beginning, but the precipitation followed soon. Due to precipitation inhibitor (PI) PVPVA and HPMC, the maximum concentration of ketoconazole improved from 1049 to 1819 and 1476 μg/mL (Figure 2A), and the major precipitation time lasted from 19 to 34 and 28 min. In the jejunal aqua phase (Figure 2B), the supersaturation and precipitation processes are similar to those in the duodenal cup. However, the pH value was relatively stable near 6, and the maximum concentration of ketoconazole of the control group, PVPVA group, and HPMC group was 282, 520, and 539 μg/mL, which was much lower than that in the duodenal cup.

The drug absorption in the gastrointestinal tract was simulated using *n*-octanol as the absorptive phase in the duodenal cup and the jejunal cup. The absorption curves in the duodenal cup absorptive phase increased slowly in the first 5 min (Figure 2C). Ketoconazole was just transported from the gastric cup to the duodenal cup. The concentration increased rapidly until 40 min. After the pumps stopped at 40 min, the increasing trend of the curves gradually slowed down. The absorption curves in the jejunal organic phase showed a similar trend, but the time was delayed by nearly 10 min, and the concentration was about twice that in the duodenal cup (Figure 2D). Because the dose of ketoconazole is large and the residence time in the duodenum is limited, most ketoconazole was further transported into the jejunum, so there was more ketoconazole in the absorptive phase of the jejunal cup. Compared with the control group, both PVPVA and HPMC promoted the absorption of ketoconazole, and the effect of PVPVA was stronger than that of HPMC. The in vitro absorption data were used to establish the in vivo and in vitro correlation models.

**Figure 3:**
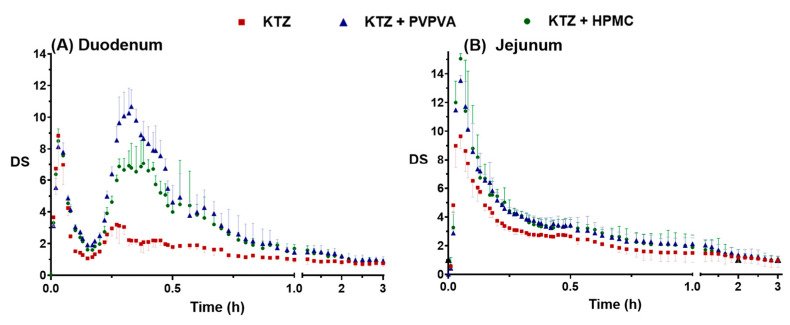
The profiles of DS–time in the simulated duodenum (**A**) and the simulated jejunum (**B**). The data points are presented as the mean ± SD (*n =* 3).

The supersaturation of ketoconazole was generated due to the pH flux during the transfer of the fluids. The process of supersaturation and the effect of precipitation inhibitor is usually evaluated by the degree of supersaturation (DS), which is defined as the ratio of the apparent concentration and the equilibrium solubility. According to the detected concentration (Figure 2) and the calculated equilibrium solubility (Figure 1), the SD–time profiles were deduced, as shown in Figure 3.

The concepts of DS_max_ (maximum degree of supersaturation) and DS_AUC_ (area under the degree of supersaturation over time curves) were utilized to provide additional insights into the “intensity” and “endurance” aspects of supersaturation, respectively. The DS_max_ and the DS_AUC_ of ketoconazole in the duodenal cup and the jejunal cup are summarized in Table 1. Ketoconazole rapidly formed supersaturation, and the DS_max_ of the three groups reached 9.64, 13.53, and 15.05, respectively, in 3 min. Then, the DS decreased rapidly because the higher DS promoted rapid precipitation. PVPVA increased the DS_max_ of ketoconazole in the duodenal aqueous phase and the jejunal aqueous phase 1.21- and 1.40-fold and increased the DS_AUC_ 1.88- and 1.26-fold, respectively. HPMC changed the DS_max_ of ketoconazole in the duodenal aqueous phase and the jejunal aqueous phase 0.96- and 1.56-fold, respectively, and increased the DS_AUC_ 1.64- and 1.22-fold, respectively. Overall, PVPVA improved and maintained the supersaturation of ketoconazole better than HPMC.

#### 2.2.2. Supersaturation and Absorption in the USP II

**Figure 4:**
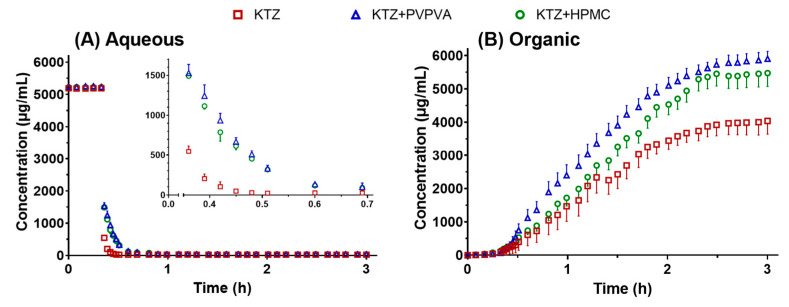
The profiles of ketoconazole concentration–time in the aqueous phase (**A**) and organic phase (**B**) in the biphasic dissolution test in USP II. The data points are presented as the mean ± SD (*n =* 3).

The concentration of ketoconazole in the aqueous phase and organic phase of USP II during the pH-shift method-induced supersaturation is illustrated in Figure 4. The concentration was immediately diluted and decreased when the concentrated FaSSIF was added and mixed with the pre-dissolved ketoconazole in the FaSSGF at 20 min. Ketoconazole precipitated quickly from 20 min to 25 min; PVPVA and HPMC inhibited the precipitation and prolonged the supersaturation to 35 min. In the organic phase, the concentration of ketoconazole increased from 20 min to over 120 min. The PVPVA and HPMC group promoted ketoconazole absorbed into the organic phase. The biphasic dissolution test in USP II has been employed in several studies to overcome the conflict between the sink condition for absorption and the non-sink condition for supersaturation [20]. The absorption data from the organic phase help the supersaturable drug delivery system achieve IVIVC.

### 2.3. Solid State Characterizations

**Figure 5:**
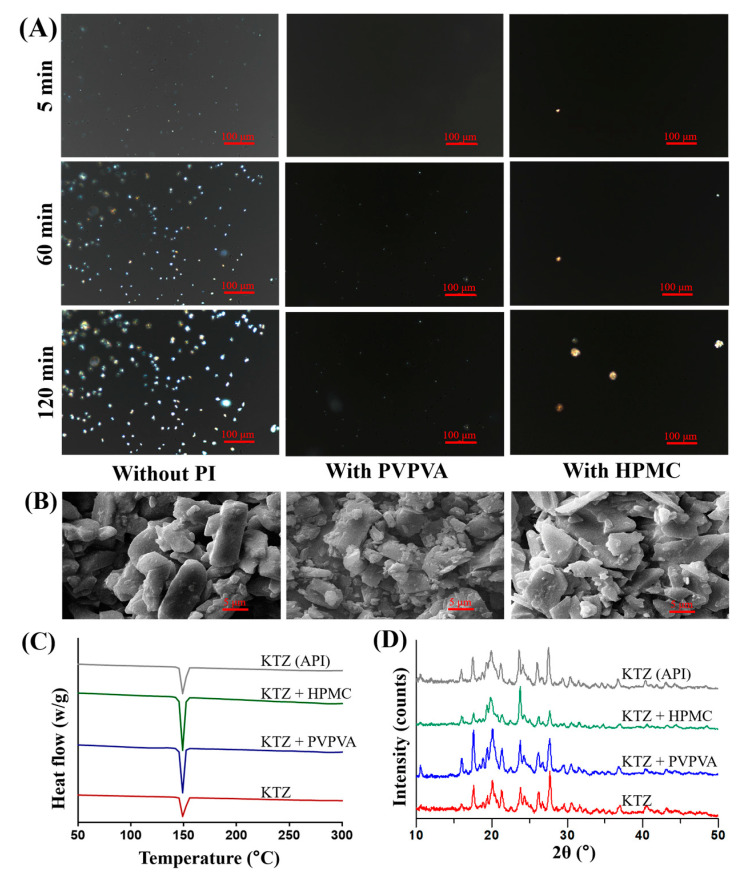
Characterization of the ketoconazole precipitation: images of polarized light microscope (**A**); Scanning Electron Microscopy (SEM) images (**B**); Differential Scanning Calorimetry (DSC) curves (**C**); and X-ray diffractograms (**D**).

The precipitation process of ketoconazole was continuously recorded by a polarized light microscope. As shown in Figure 5A, a large number of crystals was generated in the control group at 5 min. The particle size of the crystal further increased at 60 min as well as at 120 min. However, there were very few crystals in the PVPVA or the HPMC group at 5 min. The PVPVA group exhibited a limited presence of small-sized crystals for up to 60 min, and this persisted until 120 min. In the HPMC group, the number of crystals was still less, but the particle size was larger.

Scanning electron micrographs (SEM) of the ketoconazole precipitate (Figure 5B) show the microstructure of the ketoconazole precipitate. The precipitates in the control group were mainly columnar crystals and fine particles. In the PVPVA group, there were more fine particles. The precipitates in the HPMC group are mainly flake particles. The results corresponded to those of the polarizing microscope.

The Differential Scanning Calorimetry (DSC) curves (Figure 5C) show the samples processing similar endothermic peaks at 149.29, 148.46, 148.35, and 149.28 °C, respectively. The X-ray diffractograms (Figure 5D) also showed similar peaks, indicating that the precipitates of ketoconazole were in the same crystal form.

These results verify that the PVPVA and HPMC can slow down the growth of the crystal and influence the apparent morphology, but they cannot affect the crystal form of the ketoconazole precipitates. This confirms a previous in vitro study on the solid form of the ketoconazole precipitates [21].

### 2.4. Molecular Dynamic Simulation

**Figure 6:**
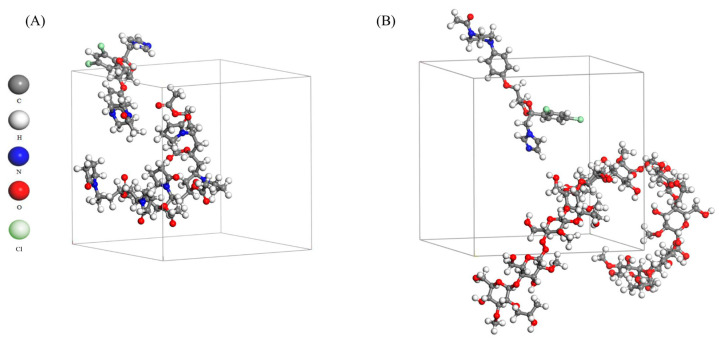
Snapshots at the end (200 ps) of the molecular dynamic simulation of (**A**) PVPVA-ketoconazole and (**B**) HPMC-ketoconazole.

The interactions between the drug molecule and the precipitation inhibitor were simulated by molecular dynamics. It provides an insight into the molecular mechanism of the drug supersaturation prolonged by PI. The equilibrium systems of ketoconazole and PVPVA/HPMC through the molecular dynamic simulation are shown in Figure 6. Intermolecular interaction energy (E_int_) is one of the main indicators reflecting the interaction between molecules. The E_int_ of PVPVA-ketoconazole and HPMC-ketoconazole were −218.403 and −178.757 kcal/mol, respectively. In addition, the van der Waals forces between ketoconazole, PVPVA, and HPMC were −266.096 and −253.586 kcal/mol, respectively. Additionally, PVPVA generated a strong electrostatic force (−557.491 kcal/mol) with ketoconazole, but HPMC did not. These results preliminarily indicate that PVPVA inhibits the aggregation of ketoconazole primarily through an electrostatic force, as well as van der Waals forces; HPMC slows down ketoconazole molecule migration mainly by van der Waals forces; and PVPVA exhibited a better effect on the supersaturation of ketoconazole due to the lower E_int_. Another study simulated that the molecular interaction between ketoconazole and Cytochrome P450 3A4 was also dominated by nonpolar van der Waals forces [22].

### 2.5. In Vivo Pharmacokinetics

**Figure 7:**
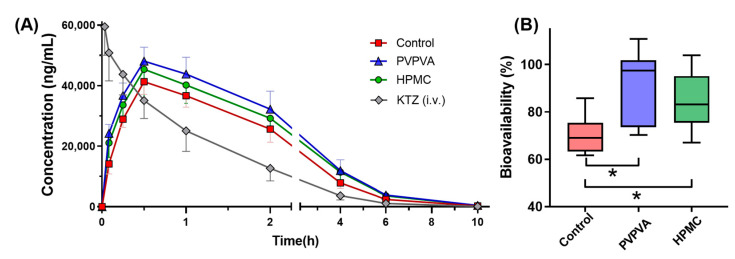
The drug plasma concentration–time profiles (**A**) and the histogram of bioavailability (**B**) of ketoconazole in each group. The data points are presented as the mean ± SD (*n =* 6); * for *p* < 0.05.

The in vivo absorption improved by supersaturation can be reflected by the pharmacokinetic parameters. As depicted in Figure 7A, both the PVPVA group and the HPMC group exhibited higher drug plasma concentration–time profiles compared to the control group. The subsequent Table 2 includes C_max_ and AUC of the drug plasma concentration–time profiles as the observed values for comparison with predicted values. PVPVA significantly improved the C_max_ 1.2-fold. The absolute bioavailability of ketoconazole was significantly increased by PVPVA and HPMC from 70% to 92% and 85% (Figure 7B). This observation implies that the presence of PVPVA and HPMC enhances the gastrointestinal absorption of ketoconazole.

### 2.6. IVIVC

**Figure 8:**
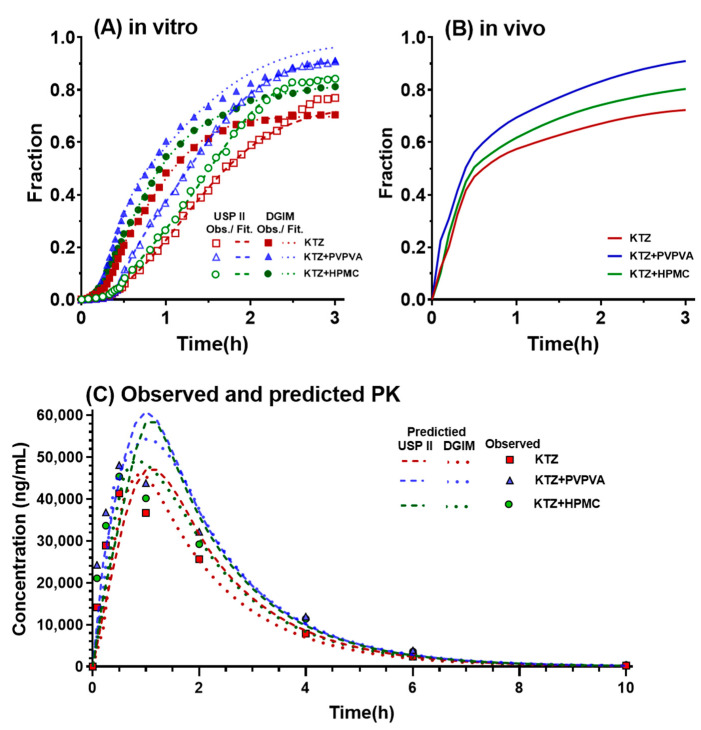
IVIVC processing DGIM vs. USPII: (**A**) in vivo fraction of absorption in DGIM vs. USP II; (**B**) in vivo fraction of absorption; and (**C**) the observed ketoconazole plasma concentration and the predicted ketoconazole plasma concentration–time profiles.

The in vitro drug-absorbed fraction (from absorption data of both the duodenum and jejunum of the DGIM) of ketoconazole and their Double Weibull Dissolution Model fitted curves in the DGIM and USP II are shown in Figure 8A. The curves in the DGIM and USP II reached a similar end, but the curves in the DGIM were ahead of the corresponding groups in USP II. The in vivo fraction of absorption (Figure 8B) was obtained from the deconvolution of the drug plasma concentration–time profiles (Figure 7A), which exhibited a similar trend as that observed for the in vitro drug-absorbed fraction. The drug dissolution and absorption were subsequently correlated using a selected mathematical model based on its lowest AIC values (DGIM: −119.4 and USP II: −142.0):

(2) Fabs=AbsScale×Diss(Tscale×Tvivo−Tshift)

where Fabs is the in vivo absorption fraction; *Diss*() is a function for dissolution, dependent on the in vitro time in the parentheses, which can be the in vivo time scaled by *Tscale* and shifted by *Tshift*; and the in vitro dissolution (*Diss*) has to be scaled by AbsScale. With the iterated final parameters, the correlation was shown as the following equations:

(3) $$Fabs(DGIM)=0.9757\times Diss(1.629\times Tvivo-4.397\times 10^{-5})$$

(4) $$Fabs(USP\;II)=0.9921\times Diss(1.835\times Tvivo-9.787\times 10^{-6})$$

Finally, the predicted plasma concentration–time profiles (Figure 8C) were obtained based on the convolution of predicted Fabs, which was calculated through the in vitro dissolution data and the above correlation. Compared with the observed ketoconazole plasma concentration at the series time points, the predicted concentration–time profiles based on the DGIM (dot lines) are closer than that based on USP II (dashed lines). Then, the prediction errors (PEs) were calculated through the percentage of deviation between the predicted value and observed value, as shown in Table 2. The mean PE of C_max_ of the groups in the DGIM is less than 10%, but that in USP II is 19.30% and over the limitation of the qualifications of IVIVC. However, both apparatuses received a mean PE of AUC within the limit (10%). This result indicates that the precipitation inhibitor-mediated supersaturation in the DGIM was well correlated to that in vivo, yet USP II failed to establish a qualified IVIVC.

Throughout this study, these results not only verified that the DGIM was more capable of achieving qualified IVIVC of the BCS IIB model drug ketoconazole than conventional USP II with an absorptive phase but also elucidated that PVPVA exhibited a better precipitation effect on ketoconazole than HPMC from the in vitro dissolution test, in vivo pharmacokinetics, and in silico molecular dynamics. The discrimination of the absorption curves between PVPVA and HPMC through the DGIM (Figure 2) was more significant than that through the USP II (Figure 4). This distinction corresponds to the in vivo absorption curves (Figure 8). The intuitional difference results in divergent IVIVC. In other words, the DGIM exhibits sufficient selectivity toward PVPVA and HPMC to effectively screen for precipitation inhibitors of ketoconazole. Molecular dynamic simulation (Figure 6) proved that PVPVA slows down ketoconazole precipitation by stronger intermolecular interaction energy than HPMC, especially by an electrostatic force.

Ketoconazole is the first Imidazole antifungal drug that can be taken orally and is widely used in superficial and systemic fungal infections [23]. Ketoconazole is also used in castration-resistant prostate cancer because it inhibits CYP17A1, which is involved in the production of sex hormones, and blocks both gonadal and adrenal steroid hormone synthesis [24]. Ketoconazole has been used as the model drug for 47 supersaturation-related studies (data from PubMed until 1 October 2023) because ketoconazole is a classical BCS IIB drug that is rather soluble in acidic gastric fluid but precipitates in the human upper intestine [25]. Different from enteric polymers, such as hydroxypropyl methylcellulose acetate succinate (HPMCAS) and Eudragit^®^ [26], pH-independent soluble PVPVA and HPMC are suitable for application in the gastrointestinal transfer-induced supersaturation of BCS IIB drugs.

There are some innated shortages of the DGIM. The surface area ratio of the jejunum and the duodenum of the absorptive phase (1:0.38) is close, but there exists a gap in the physiological parameters (1:0.49) [27] because the DGIM employed a standard dissolution cup (diameter 102 mm) for the jejunal cup and a small dissolution cup (diameter 62 mm) for the duodenal cup. A solution is to custom-make a dissolution cup with a diameter of 70 mm. Another limitation is that the ileum and other lateral regions of the intestine were omitted to balance the precision and concision. Though the ileum also presents a large surface area for drug absorption, the immediate-release supersaturable formulations are mainly absorbed in the duodenum and jejunum. For the sustained-release preparations, an additional cup for the ileum may be important for the IVIVC.

Model-informed drug development is not only explored by academics and the industry but also encouraged by regulations, including from the U.S. Food and Drug Administration and China National Medical Products Administration [28,29]. IVIVC is one of the most commonly used models, which helps to provide regulatory evidence for changes in scale-up and post-approval changes, to set dissolution specifications, and to obtain biowaivers, usually referring to parts of a larger framework, such as Quality by Design, life-cycle management, or a drug formulation strategy. According to a survey on IVIVC development in the pharmaceutical industry, enhanced alternative dissolution methods are expected for a more in-depth evaluation of the physiological factors that affect in vivo dissolution within IVIVC development [30]. For BCS II drugs with high permeability but poor solubility, the concentration in the gastrointestinal tract limits absorption. The pH-dependent soluble weakly basic drugs of the BCS class II are affected by the pH shift during transport through the gastrointestinal tract.

Previous research [7,8,9,12] developed and applied a gastrointestinal simulator (GIS) to simulate transportation, the pH shift, and some other physiological conditions. They focused on the supersaturation and precipitation curves in the intestinal fluids. Their IVIVCs were built based on the dissolution curves in the intestinal fluids or a combined in situ mouse infusion, usually correlating the C_max_ or AUC for Level B or Level C IVIVCs. This study developed the dynamic gastrointestinal model (DGIM) as a physiologically based multi-cup dissolution method to simulate gastrointestinal transportation. In addition, it combined organic phase-simulated absorption, which provided better in vitro data to achieve Level A IVIVC. The results tentatively demonstrate the capability of the DGIM to facilitate the implementation of IVIVC for the BCS IIB model drug ketoconazole. Additional cases are required to further substantiate the capabilities of the DGIM.

Future research can develop both the mathematical model and the instrumental model by some emerging technologies, such as artificial intelligence (AI), microfluidic chip-based organoids, and digital twins. AI has been employed in some pharmaceutical research [31], including dissolution tests, but is limited to some regular formulations [32]. AI can bypass the limitations of current mathematical models and predict in vivo absorption based on big data, including the previous dissolution and properties of the components and excipients. On the other hand, with the progress of organoids, many intestinal organoid models have been developed and used to study intestinal physiology and develop drugs [33], including drug absorption-related nephrotoxicity [34] and drug absorption-related pharmacokinetics and pharmacodynamics [35]. Hence, there is a small gap for microfluidic chip-based intestinal organoids to be an advanced alternative to instrumental models. With specially designed channels and intestinal organoids on a microfluidic chip, the gastrointestinal tract can be better simulated for evaluating the supersaturation of BCS IIB drugs in vitro and contributing to successful IVIVC. In addition, digital twins are an emerging virtual reality technology that have recently been introduced in the pharmaceutical industry for their cost-effectiveness and time efficiency [36]. Recently, a pioneer employed a digital twin to simulate the colon [37] and further virtually mimic the dissolution of a tablet in the colon [38]. Therefore, it is feasible to construct a digital twin of the drug formulation and gastrointestinal tract in order to simulate the dissolution, supersaturation, and precipitation influenced by a precipitation inhibitor in silico.

## 3. Materials and Methods

### 3.1. Materials

Ketoconazole (99.8% purity) was from Shanghai Aladdin Reagent Co., Ltd. (Shanghai, China). Hydroxypropyl methylcellulose (HPMC) was generously provided by Colorcon Ltd. (Dartford, UK). Polyvinylpyrrolidone-vinyl acetate copolymer (PVPVA) was kindly gifted by BASF Ltd. (Ludwigshafen, Germany). Fasted Simulated Intestinal Fluid (FaSSIF) powder was from biorelevant.com Ltd. (London, UK). Chromatographic pure acetonitrile, methanol, phosphoric acid, and potassium dihydrogen phosphate were from Sigma-Aldrich Co., Ltd. (Saint Louis, MO, USA). Other reagents were purchased from Sinopharm Chemical Reagent Co., Ltd. (Shanghai, China) unless otherwise stated.

### 3.2. pH-Dependent Solubility Measurements

The equilibrium solubility of ketoconazole is influenced by the pH value and composition of the solution medium. The equilibrium solubility was determined in various FaSSIF solutions with adjusted pH at 2, 3, 4, 4.7, 5, 6, and 6.5, with the PI or not. Specifically, FaSSIF was prepared with different proportions of 0.2 mol/L disodium hydrogen phosphate (Na_2_HPO_4_) solution and 0.1 mol/L citric acid (C_6_H_8_O_7_) solution, with commercial standard FaSSIF powder. In addition, the buffer for the PVPVA group and the HPMC group was composed of 10 mM PVPVA and HPMC, respectively. Then, slightly excessive ketoconazole was weighted into 5 mL Eppendorf tubes along with 2 mL of the respective buffer solutions mentioned above, followed by shaking at 37 °C for a duration of 24 h. The final pH of each solution was measured before centrifugation at 10,000× *g* for 10 min at 37 °C. The resulting supernatant was then diluted to 1/100 (for samples with pH values of 2 and 3), 1/10 (for samples with pH values of 4, 4.7, and 5), and 1/2 (for samples with pH values of 6 and 6.5) with a mixture consisting of acetonitrile and water in a ratio of 50:50 for analysis using high-performance liquid chromatography (HPLC).

The Agilent 1260 Infinity HPLC (Agilent Technologies, Santa Clara, CA, USA) was utilized to determine the equilibrium solubility of ketoconazole in dissolution experiments with different PI and pH. A sample volume of 10 μL was injected and subsequently separated on a Kromasil C18 column (5 μm, 4.6 × 250 mm) maintained at a temperature of 30 °C. The mobile phase consisted of a modified phosphoric acid solution containing 10 mM KH_2_PO_4_ in water (pH adjusted to 3.0), along with acetonitrile in a ratio of 50:50 (*v*/*v*). The flow rate employed was set at 1 mL/min while detection occurred at a wavelength of 254 nm.

### 3.3. In Vitro Supersaturation–Precipitation and Absorption Assay

#### 3.3.1. Supersaturation–Precipitation and Absorption in the DGIM

**Figure 9:**
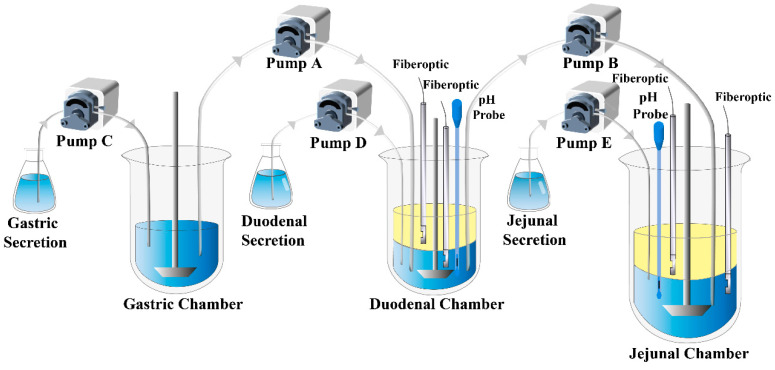
The schematic of the dynamic gastrointestinal model (DGIM).

The DGIM is achieved through a system consisting of 3 dissolution cups, which represent stomach, duodenum, and jejunum, respectively, filled with corresponding biorelevant fluid. *N*-octanol serves as the absorptive phase. The pumps facilitate fluid transfer, as depicted by arrows in Figure 9. In situ drug concentration is determined using fiber optical probes, and dynamic pH value of the aqueous fluid is monitored by pH electrodes. The parameters of DGIM can be adjusted to meet some special physiological conditions.

In this study, most of the parameters were based on the GIS proposed by Hens et al. [39]. The pH values in the three cups were adjusted to 2.0, 5.9, and 6.1, respectively, to simulate the physiological digestive fluid in the stomach, duodenum, and jejunum of the fasted rats [40]. The first dissolution cup representing the stomach initially contained 300 mL of simulated gastric juice ($V_{g,0}$), containing ketoconazole at a concentration of 5.2 mg/mL and physically mixed PVPVA/HPMC at a concentration of 10 mg/mL (PVPVA group and HPMC group), or containing no additives (control group); the second dissolution cup representing duodenum initially contained 50 mL simulated intestinal juice ($V_{d,0}$) along with 100 mL octanol; and the third dissolution cup representing jejunum initially contained 100 mL simulated intestinal juice ($V_{j,0}$) and the same 100 mL of octanol. The secretions used were biorelevant fluids that had been concentrated four times and pumped at a rate of 1 mL/min. Considering an average gastric half-emptying time ($t_{h}$) of approximately eight minutes, pump A transported gastric fluid into the duodenal cup at this first-order rate. Ensuring that the volume of biorelevant fluid in the duodenal cup was maintained at a constant level of 50 mL, any excess fluid was transported to the jejunal cup through pump B. Therefore, we can represent the rates $v_{a}$ for pump A and $v_{b}$ for pump B at time t using following equations:

(5) $$v_{a}=ln\left(2\right)\times V_{g,0}\times exp\left(-\left(ln\left(2\right)\times t/t_{h}\right)\right)+1$$

(6) $$v_{b}=v_{a}+1$$

The pump rate was regulated by a mono-chip computer, which was programmed to dynamically adjust the rate every second in accordance with first-order kinetics. This approach is more accurate than the combination of zero-order kinetic rates to mimic first-order-like kinetics, as described by Yasuhiro et al. [10] in their work on GIS-α. The pumps worked until 40 min because, at that time, the solution in the gastric cup was less than 10 mL. However, the experiment continued for 3 h. The paddles were stirred at a rate of 50 rpm. All the cups were in a water bath at 37 °C.

The Rainbow^®^ UV-Vis fiber optic instrument (Pion Inc., Billerica, MA, USA) was utilized to continuously monitor the concentration of ketoconazole. Fiber optic probes with a 1 mm tip were connected to the instrument and employed for the detection of the 270–275 nm wavelength band. This detection specifically targeted the area under the curve (AUC) of the second derivatized absorbance curves. Calibration was performed using the accumulated addition method. The standard curves encompassed a wide concentration range in the dissolution process while ensuring linearity with an r^2^ > 0.999.

#### 3.3.2. Supersaturation–Precipitation and Absorption in the USP II

The supersaturation in DGIM was compared using a conventional pH-shift method conducted in USP apparatus II (paddles) at 37 °C and 50 rpm. Ketoconazole was pre-dissolved in 250 mL FaSSGF at a concentration of 5.2 mg/mL, with the addition of either 10 mg/mL PVPVA or HPMC (in the PVPVA group or HPMC group) or without any additives (in the control group). Subsequently, 200 mL of mutually saturated *n*-octanol was carefully introduced into the aqueous phase. After a duration of 20 min, 500 mL of a concentrated FaSSIF solution (1.5-fold dilution) was cautiously poured into the dissolution cup. Two fiber optic probes were employed to continuously monitor ketoconazole concentrations in both the aqueous and organic phases for a period of three hours.

### 3.4. Solid State Characterizations

#### 3.4.1. Polarizing Microscopy

To observe the crystal growth of ketoconazole under various precipitation conditions, a Nikon Ti-E microscope, fitted with a digital camera DS Fi1 manufactured by Nikon in Tokyo, Japan, was utilized. The supersaturated solution was induced using the pH-shift method and subsequently applied as a single droplet onto a single concave glass slide placed on a temperature-controlled platform set to 37 °C. The photos were recorded after intervals of 5, 60, and 120 min using objective lenses with a magnification factor of 20× under polarized light.

#### 3.4.2. Scanning Electron Microscopy

To detect the microstructure of drug precipitates, the precipitates were collected from the jejunal cup after the above dissolution test and subsequently dried overnight at 37 °C (similarly for the following two tests). The dried samples were mounted on a copper stub, respectively, and then coated with gold under vacuum. The micromorphology and microstructure of the precipitates were observed and captured by a JSM-6510 scanning electron microscope manufactured by JEOL in Tokyo, Japan, with an acceleration voltage of 10–15 kV.

#### 3.4.3. X-ray Powder Diffraction

To analyze the crystal structure of the precipitates, a BrukerD8 X-ray powder diffractometer manufactured by Bruker in Bremen, Germany, was used. The generator of the Cu-Kα radiation source was set at 40 mA and 40 kV, and the angular resolution was set at 0.02° (2θ), with the detector opening set at 4.85° and each step taking approximately 45 s for measurement.

#### 3.4.4. Differential Scanning Calorimetry

To investigate the solid state of the precipitates, a Mettler Toledo DSC3+ manufactured by Mettler Toledo in Greifensee, Swiss, was utilized. The precipitates were put in standard aluminum pans and subjected to heating at a rate of 10 °C/min from 25 to 300 °C under an atmosphere of dry nitrogen.

### 3.5. Molecular Dynamic Simulation

The molecular interactions between ketoconazole and PVPVA or HPMC were simulated by Materials Studio (Accelrys, San Diego, CA, USA). The ratio of ketoconazole to PVPVA and HPMC segments was 1:5. The molecules were conducted geometrically optimized through the COMPASS force field, with the cascade of steepest descents, convoluted gradients, and quasi-Newtons. Then, the molecular dynamics simulation processed the Dynamic task under the Forcite module with the constant volume and temperature (NVT) ensemble. The electrostatic and van der Waals effects were calculated using Ewald and Atom-based methods, with a time step of 1 fs and a total simulation duration of 200 ps at 310 K by Nose temperature control method. The distribution and intermolecular interaction energy were obtained from the equilibrium system with the lowest energy.

### 3.6. Pharmacokinetic Study

The animal study was conducted in accordance with the National Research Council’s Guide for the Care and Use of Laboratory Animals and approved by the Ethics Committee of Chengdu University of Traditional Chinese Medicine (Approval Code: 2021-76). All procedures were performed in compliance with relevant regulations and guidelines. Male Sprague Dawley rats weighing 180–220 g were fasted overnight (12 h) with free access to water, then randomly assigned to 4 groups (*n =* 6). The drug was dissolved in a solution with a pH value of 2.0 at a concentration of 1 mg/mL without additional precipitation inhibitors. The control group received oral administration of ketoconazole at a dose of 10 mg/kg, while the PVPVA and HPMC groups were orally administered the same dosage along with an additional 10 mg/mL of PVPVA or HPMC in the solution, respectively. The last group was intravenously injected through the caudal vein with ketoconazole administered at a dose of 2.5 mg/kg (0.5 mg/mL ketoconazole in a solution containing propylene glycol and normal saline at a ratio of 3:2 (*v*/*v*)). Approximately 50 μL of blood samples were collected into EDTA-K_2_-treated Eppendorf tubes at time points ranging from 0.083, 0.25, 0.5, 1, 2, 4, and 6 to 10 h after dosing. The blood samples were centrifuged at 2000× *g* and 4 °C for 10 min, followed by collection and storage of the supernatant plasma at −20 °C until analysis.

The concentration of ketoconazole in plasma was determined using liquid chromatography-tandem mass spectrometry (LC-MS), which consisted of a Shimadzu LC-30A HPLC and an Applied Biosystems/MDS SCIEX QTRAP 5500 mass spectrometer. A protein precipitation method was employed to pretreat 10 μL plasma with 430 μL acetonitrile, 50 μL internal standard solution (gefitinib), and 10 μL mobile phase. After centrifugation at 10,000× *g* for 10 min at 4 °C, the supernatant was injected into the apparatus and then separated by a Thermo Hypersil C18 chromatographic column (3 μm, 2.1 mm × 50 mm) at 40 °C. The mobile phase consisted of an aqueous formic acid solution containing ammonium acetate (5 mM) and acetonitrile in a ratio of 50:50% (*v*/*v*), with a flow rate of 0.4 mL/min for each sample lasting for approximately 1.5 min. The positive ion mode of the mass spectrometer was utilized, employing specific mass transitions to ketoconazole of *m*/*z*531.2→489.3 and to internal-standard gefitinib of *m*/*z*447.1→128.1. Phoenix 8.1 WinNonlin Toolkit (Certara, Princeton, NJ, USA) was employed to conduct the non-compartment model of pharmacokinetic analysis.

### 3.7. In Vitro–In Vivo Correlation

The in vitro–in vivo correlation (IVIVC) was performed using Phoenix 8.1 IVIVC Toolkit (Certara, Princeton, NJ, USA). The dissolution data of ketoconazole in organic phases within the duodenal and jejunal compartments of the DGIM were combined and utilized as the in vitro absorption data. Double Weibull Dissolution Model was selected for fitting both dissolution data from the absorptive phase of DGIM and USP II. The absorption data were obtained via deconvolution analysis of the drug plasma concentration–time curves. The reference formulation for deconvolution to estimate oral absorption was based on the in vivo data of the i.v. group. Additionally, both control group and PVPVA group’s in vitro data were used to establish a correlation between dissolved fraction and absorbed fraction, while the HPMC group’s data served as external validation. The maximum number of unit impulse response exponentials defaulted to three. With WinNonlin Generated Initial Parameter and Winnonlin Bounds, the best correlation model was selected according to the lowest AIC. Validation parameters included AUC_last_ calculated with linear trapezoidal method along with linear interpolation averaging as mean values were adopted. The process of prediction followed the same parameters above. The prediction error (PE) was evaluated following the FDA’s guidance [41]. For comparison purposes, ketoconazole dissolution data from the absorptive phase of USP II were also employed as in vitro absorption data using a similar process for establishing IVIVC relationship. Absorption measurements commenced 20 min after pH-shift operation.

### 3.8. Statistics

GraphPad Prism software version 8 (GraphPad Software, Inc., San Diego, CA, USA) was utilized for curve fitting and statistical analyses. Student’s *t*-test and one-way analysis of variance were employed for group comparisons. The results were presented as mean ± standard deviation (SD). Statistical significance was denoted by asterisks: * for *p* < 0.05, ** for *p* < 0.01, and *** for *p* < 0.001.

## 4. Conclusions

A multi-cup dissolution approach named the dynamic gastrointestinal model (DGIM) was developed based on USP apparatus II (paddle method) in this study, and its superior in vivo predictability compared to the conventional USP II method for BCS IIB drug ketoconazole in the case study was confirmed through IVIVC. This inherent versatility of the DGIM enables easier implementation in the pharmaceutical industry. Utilizing the DGIM in the case study, it was elucidated that both PVPVA and HPMC can enhance and sustain ketoconazole supersaturation, leading to increased in vitro drug absorption that aligns with the observed in vivo absorption trend. Molecular dynamic simulation revealed that PVPVA exhibits a more effective inhibitory effect on ketoconazole molecule aggregation than HPMC, attributed not only to slightly higher van der Waals forces but also a unique electrostatic force. In summary, the DGIM offers a promising approach for simulating physiological conditions of the gastrointestinal tract to improve the evaluation of BCS IIB drugs with supersaturation potential, facilitating the establishment of IVIVC and identification of precipitation inhibitors.

## Figures and Tables

**Table 1:** The DS_max_ and the DS_AUC_ affected by precipitation inhibitors.

Group	Duodenal		Jejunal	
	DS_max_	DS_AUC_	DS_max_	DS_AUC_
Control	8.837	3.715	9.643	5.452
PVPVA	10.68	6.988	13.53	6.859
HPMC	8.507	6.095	15.05	6.660

**Table 2:** Prediction errors (PE) of DGIM and USP II based on the IVIVC.

		DGIM				USP II			
		Control	PVPVA	HPMC	Mean	Control	PVPVA	HPMC	Mean
C_max_ (ng·mL^−1^)	Observation	41,300	48,100	45,400		41,300	48,100	45,400	
	Prediction	45,529	54,269	48,488		45,702	58,480	57,044	
	PE (%)	10.24	12.83	6.80	9.96	10.66	21.58	25.65	19.3
AUC (h·ng·mL^−1^)	Observation	112,853	146,143	135,015		112,853	146,144	135,015	
	Prediction	114,652	144,456	135,032		108,727	149,731	126,032	
	PE (%)	1.59	−1.16	0.01	0.92	−3.66	2.45	−6.65	4.25

## Data Availability

The data obtained for the publication of this article are available upon reasonable request to the corresponding author.

## Sample Availability

Available.
